# Temporal and spatial variation characteristics of environmental iodine elements in the Gansu region, China

**DOI:** 10.3389/fnut.2026.1915926

**Published:** 2026-10-08

**Authors:** Yongqin Cao, Yating Jin, Wei Sun, Yugui Dou, Yanling Wang, Ye Ruan

**Affiliations:** 1 Department of Endemic Disease Prevention and Control, Gansu Provincial Center for Disease Control and Prevention, Lanzhou, China; 2 School of Public Health, Lanzhou University, Lanzhou, China

**Keywords:** iodine deficiency, environmental monitoring, spatiotemporal analysis, hydrological systems, Gansu Province

## Abstract

**Objective:**

This study investigates the spatiotemporal variation of iodine concentrations in drinking water and soil across Gansu Province, aiming to generate observational evidence for adjusting iodine deficiency disorder (IDD) prevention strategies, given inherent methodological limitations of historical unpaired cross-sectional surveys.

**Methods:**

We collected data on water iodine from four surveys (1972, 1985, 2014, and 2017) and soil iodine from two surveys (1985 and 2014) in Gansu Province, along with related information. These data were analyzed to examine trends in iodine levels across different time periods, watersheds, and geomorphological types.

**Results:**

Water iodine concentrations consistently followed the order: Yellow River > Inland River > Yangtze River at the watershed level, and Longdong > Longzhong > Hexi > Longnan at the regional level, with consistent distribution patterns across both watersheds and regions.Water iodine concentrations exhibited temporal fluctuations across survey rounds, with relatively lower iodine medians observed in recent surveys; these apparent shifts may partly stem from sampling heterogeneity rather than genuine environmental change. However, due to the unpaired sampling design and methodological variability across surveys, these findings should be interpreted as observational patterns rather than definitive causal trends.Soil iodine content in Hexi and Longnan decreased significantly, and soil iodine content in other regions did not change significantly.

**Conclusions:**

Persistent environmental iodine deficiency was observed across all surveys in Gansu's hydrological systems, with temporal fluctuations that suggest potential changes warranting further investigation.The pronounced reduction of topsoil iodine observed in arid inland basins may indicate possible shifts in biogeochemical cycling, although analytical or sampling artifacts cannot be fully excluded. Critical knowledge gaps persist regarding atmospheric iodine fluxes, necessitating integrated air-water-soil monitoring systems to elucidate complete iodine ecodynamics. Our observational findings underline that future paired-site monitoring, including atmospheric iodine measurements, is required to fully disentangle regional environmental iodine cycling.

## 1 Introduction

Iodine is an essential trace element for human thyroid hormone synthesis, and its environmental availability directly influences the iodine nutritional status of populations. Globally, approximately 1.9 billion people live in iodine-deficient areas, with endemic goiter and iodine deficiency disorders (IDD) remaining major public health challenges in many developing regions (1, 2). The biogeochemical cycle of iodine—involving atmospheric, terrestrial, and aquatic reservoirs—determines the baseline iodine concentration in drinking water and soil, which in turn shapes the iodine intake of local residents (3). However, this natural cycle is increasingly perturbed by climate change, land-use alteration, and hydrological modifications, making it critical to understand long-term environmental iodine dynamics for evidence-based IDD prevention policies (4).

The relationship between climate and environmental iodine is complex and bidirectional. Precipitation, as a primary driver of the iodine cycle, can simultaneously increase riverine iodine concentrations by leaching iodine from rocks and soils into surface waters, while also accelerating the loss of soil iodine through vertical leaching (5, 6). Temperature rise further complicates this picture by enhancing volatilization of iodine from water bodies into the atmosphere and increasing glacial meltwater fluxes, which may dilute iodine in downstream aquatic systems (7). Despite these recognized mechanisms, empirical data linking long-term climate trends with environmental iodine changes remain scarce, particularly in arid and semi-arid regions where water scarcity already poses significant challenges (8).

Gansu Province in northwestern China presents an ideal natural laboratory for investigating long-term environmental iodine dynamics. The province encompasses three major river basins (Yellow River, Inland River, and Yangtze River) and four distinct geomorphological regions (Hexi, Longzhong, Longdong, and Longnan), spanning a wide gradient of precipitation, temperature, and vegetation cover (9, 10). Critically, Gansu has maintained systematic water iodine surveys since 1972 and soil iodine surveys since 1985, generating a unique 45-year longitudinal dataset that is exceptionally rare in iodine ecology research. Moreover, the Yellow River Basin within Gansu contributes nearly one-third of the annual sediment load to the lower reaches (11), making this region particularly sensitive to environmental changes that may affect iodine distribution.

In this study, we aimed to (1) characterize the spatiotemporal variation of iodine concentrations in drinking water and soil across Gansu Province over the 1972–2017 period; (2) examine the consistency between watershed-level and region-level iodine distributions; and (3) explore the potential environmental drivers—including precipitation, temperature, and vegetation—underlying the observed trends. Our findings are expected to provide critical baseline data for refining regional IDD prevention strategies and for identifying knowledge gaps in the atmospheric iodine component of the biogeochemical cycle.

## 2 Materials and methods

### 2.1 Source

Residential drinking water iodine surveys in 1972 (6), 1985 (7), 2014 (8), and 2017 (9).

### 2.2 Sample collection and analytical procedures

The water and soil iodine data analyzed in this study were derived from four provincial surveys (water: 1972, 1985, 2014, 2017; soil: 1985, 2014) conducted by the Gansu Provincial Center for Disease Control and Prevention. The sampling protocols varied across survey years due to the historical nature of the datasets, and we describe each below.

In the 1972 survey, water samples were collected from major drinking water sources (including deep wells, rivers, and tap water) across all counties in Gansu, following the military hygiene survey protocol (6). The 1985 survey adopted a stratified sampling design based on administrative counties, with samples collected from representative residential drinking water sources (7). The 2014 and 2017 surveys followed the national standardized protocol for drinking water iodine investigation, using a township-level sampling framework to ensure comprehensive geographic coverage (8, 9). In all surveys, water samples were collected in acid-washed polyethylene bottles, stored at 4 °C, and analyzed within 48 h of collection. Some methodological metadata from the earliest 1972 and 1985 surveys are incomplete in archived documents. While we retrieved all available protocol records, subtle analytical drift across decades cannot be completely excluded.

Soil samples were collected from the topsoil layer (0–20 cm) using a five-point sampling method within each designated sampling unit in 1985 and 2014. Each composite sample consisted of five subsamples thoroughly mixed to represent the spatial variability of the sampling site. Soil samples were air-dried, ground, and sieved through a 100-mesh (0.149 mm) nylon sieve prior to analysis.

Water iodine concentration was determined using the arsenic-cerium catalytic spectrophotometric method (based on the national standard GB/T 5750.5-2006 for recent surveys, and its comparable predecessor for historical data). Soil iodine was determined by pyrolysis alkaline ashing followed by arsenic-cerium catalytic spectrophotometry, consistent with the national standard method (GB/T 19380-2016 for recent surveys). The limit of detection (LOD) was 0.5 μg/L for water iodine and 0.1 mg/kg for soil iodine. For samples with concentrations below the LOD, a value of LOD/2 was assigned for statistical analysis. Assigning LOD/2 for values below the detection limit is standard practice; potential impacts of this substitution on median estimates are addressed in the limitation section.

Throughout all surveys, national certified reference materials (GBW series, including GBW 09139 for water and GBW 07401 for soil) were analyzed in each batch to validate analytical accuracy. Duplicate samples (10% of total samples) and reagent blanks were routinely included to ensure reproducibility and to monitor potential contamination. Inter-laboratory calibrationon exercises were conducted prior to each survey to ensure consistency across participating laboratories.

We acknowledge that the sampling sites were not strictly paired across the different survey years, as the surveys were designed independently for cross-sectional monitoring rather than longitudinal tracking. This methodological constraint limits our ability to attribute observed changes solely to environmental factors, and we have therefore framed our findings as observational spatiotemporal patterns rather than definitive causal trends. We discuss the implications of this limitation in Section 4.5. All surveys covered the same administrative units (county or township level) within each respective region, ensuring geographic comparability across survey periods despite variations in sample density.

Sample sizes varied substantially across years, and sampling density heterogeneity may influence observed median values. We cannot fully rule out potential systematic bias caused by uneven spatial coverage. Sensitivity considerations for uneven sample sizes are further discussed in Section 4.5 Limitations.

### 2.3 Watershed and regional division

The rivers in Gansu Province are divided into three major basins, namely the Yangtze River, the Yellow River, and the Inland River, according to their destinations (10). Gansu has a diverse range of ecosystem types and classifications. Based on the distribution characteristics of landforms, the entire province is divided into four areas: Hexi, Longzhong, Longdong, and Longnan (7).

The Hexi region is located in the northwestern part of Gansu, bordering the Qilian Mountains to the south and the Inner Mongolian Plateau to the north; it forms a long, narrow corridor (the Hexi Corridor) with an arid climate. The Longzhong region occupies the central part of the province, centered around Lanzhou and Dingxi, with a transitional climate between semi-arid and semi-humid. The Longdong region lies in the eastern part of the province, encompassing the Qingyang and Pingliang areas, characterized by the Loess Plateau terrain. The Longnan region is situated in the southern part of Gansu, including Longnan and Gannan, with a humid to semi-humid climate and mountainous terrain (10).

### 2.4 Statistical methods

An Excel database was established, and statistical analysis was conducted using SPSS 26.0 (IBM Corp., Armonk, NY, USA). Data normality was assessed using the Kolmogorov–Smirnov test; all iodine concentration data were found to be non-normally distributed (*P* < 0.05). Iodine concentrations were therefore summarized as medians with 25th and 75th percentiles (P25–75). For comparisons between two independent samples, the Mann–Whitney *U*-test was applied; for comparisons among multiple independent samples (across years, watersheds, or regions), the Kruskal–Wallis *H*-test was used. To control for Type I error inflation due to multiple comparisons, Bonferroni correction was applied for omnibus overall tests. The significance threshold was set at α = 0.05 (two-sided) after Bonferroni adjustment.

For this study, only aggregated summary outputs from historical cross-sectional surveys are available, without individual-level raw records under institutional data-protection constraints. Therefore, software-generated effect-size metrics (Cohen's d, eta-squared) and bootstrap-based 95 % confidence intervals for medians cannot be computed. Raw pairwise *post-hoc P*-values for individual intergroup comparisons are also unavailable from archived materials. Only omnibus global test statistics, including χ^2^, Z, and Bonferroni-adjusted omnibus *P*-values, are presented in the corresponding tables.

## 3 Results

Figure 1 Geographic map of the study area in Gansu Province, showing the three major river basins (Yellow River, Inland River, and Yangtze River) and the four geomorphological regions (Hexi, Longzhong, Longdong, and Longnan) included in this analysis. Base map sourced from the Gansu Provincial Standard Map Online Service System [GS (2023) 31, GS (2025) 163].

**Figure 1:**
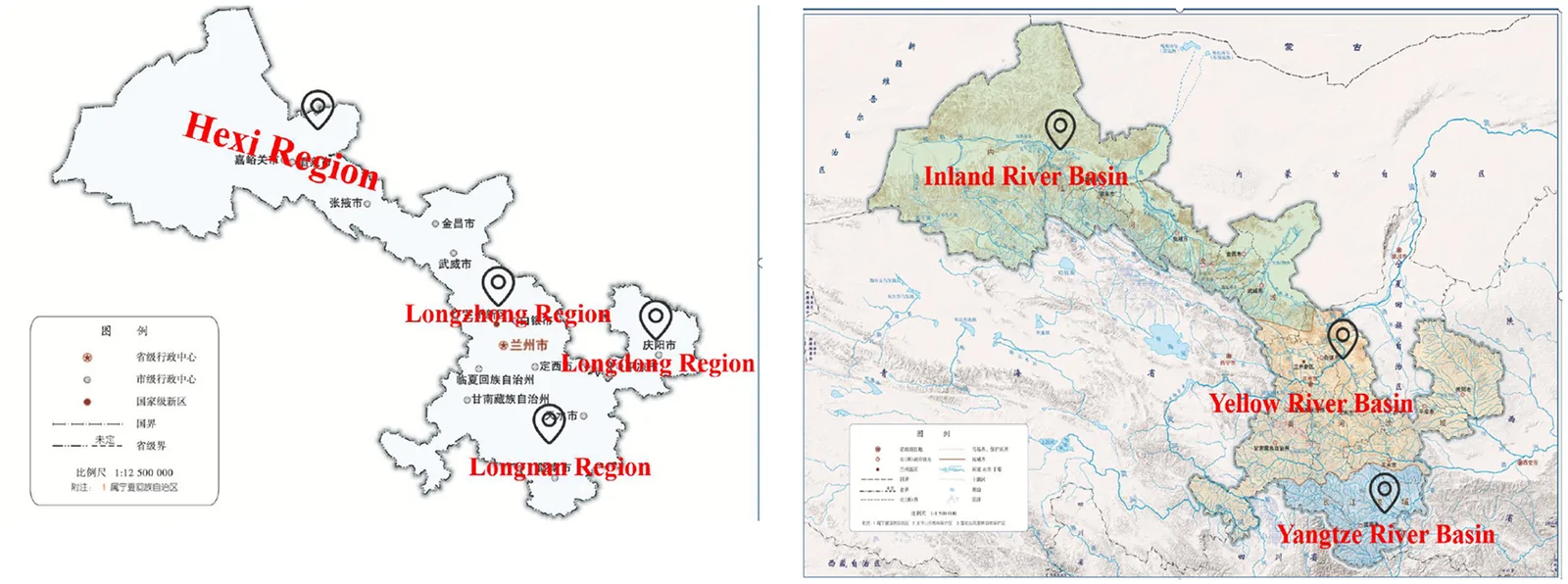
Geographic map of the study area in Gansu Province. This figure uses a base map sourced from the Gansu Provincial Standard Map Online Service System (GS(2023)31, GS(2025)163).

### 3.1 Water iodine distribution in Gansu

#### 3.1.1 Water iodine levels in different watersheds

Table 1 shows the median water iodine levels in inland river basins from the results of four tests in 1972, 1985, 2014, and 2017 were 4.0, 3.6, 1.6, and 1.6 μg/L, respectively, The median water iodine values in the Yellow River basin were 8.0, 6.1, 3.0 and 2.8 μg/L, respectively, and in the Yangtze River basin (no data in 1972) were 1.7, 1.9 and 1.4 μg/L, respectively; the iodine content of water in the inland river basin showed statistically significant differences across the four survey years (*P* < *0.05*, after Bonferroni correction; see Table 1 footnotes for detailed test statistics). However, we caution that these differences do not necessarily represent a continuous temporal decline, as the sampling sites were not paired across surveys, and the magnitude of observed differences followed the order: Yellow River > Inland River > Yangtze River. Water iodine concentrations showed significant differences across the four survey years within each of the three watersheds (all *P* < 0.05 after Bonferroni correction; see Table 1 footnotes for detailed test statistics). Across the same survey periods, water iodine levels also differed significantly among the three watersheds (all *P* < 0.05), consistently following the order: Yellow River > Inland River > Yangtze River (Figure 2).

**Table 1:** Comparison of water iodine content in different watersheds.

Watershed	Year	Number of copies	M (*P*_25_ ~*P*_75_) (μg/L)	Iodine frequency distribution [***n*** (%)]					Significance test	
				0~	10~	20~	30~	40~	χ^2^	*P*
Inland river	1972	1835	4.0 (2.0 ~ 8.0)	1525 (83.1)	222 (12.1)	40 (2.2)	17 (0.9)	31 (1.7)	67.677	0.000
	1985	49	3.6 (2.7 ~ 6.6)	42 (85.7)	5 (10.2)	2 (4.1)	0 (0.0)	0 (0.0)		
	2014	300	1.6 (0.5 ~ 13.5)	217 (72.3)	17 (5.7)	65 (21.7)	0 (0.0)	1 (0.3)		
	2017	232	1.6 (0.7 ~ 3.2)	229 (98.7)	3 (1.3)	0 (0.0)	0 (0.0)	0 (0.0)		
Yellow river	1972	1316	8.0 (4.0 ~ 10.0)	782 (59.4)	465 (35.3)	11 (0.8)	7 (0.5)	51 (3.9)	310.661	0.000
	1985	81	6.1 (2.7 ~ 10.9)	58 (71.6)	18 (22.2)	3 (3.7)	1 (1.2)	1 (1.2)		
	2014	654	3.0 (0.7 ~ 10.6)	489 (74.8)	55 (8.4)	16 (2.4)	93 (14.2)	1 (0.2)		
	2017	805	2.8 (1.4 ~ 5.0)	746 (92.7)	46 (5.7)	6 (0.7)	3 (0.3)	4 (0.5)		
Yangtze river	1985	24	1.7 (1.3 ~ 2.7)	24 (100.0)	0 (0.0)	0 (0.0)	0 (0.0)	0 (0.0)	14.598	0.001
	2014	247	1.9 (0.8 ~ 4.0)	233 (94.3)	13 (5.3)	0 (0.0)	1 (0.4)	0 (0.0)		
	2017	148	1.4 (0.7 ~ 2.4)	148 (100.0)	0 (0.0)	0 (0.0)	0 (0.0)	0 (0.0)		

**Figure 2:**
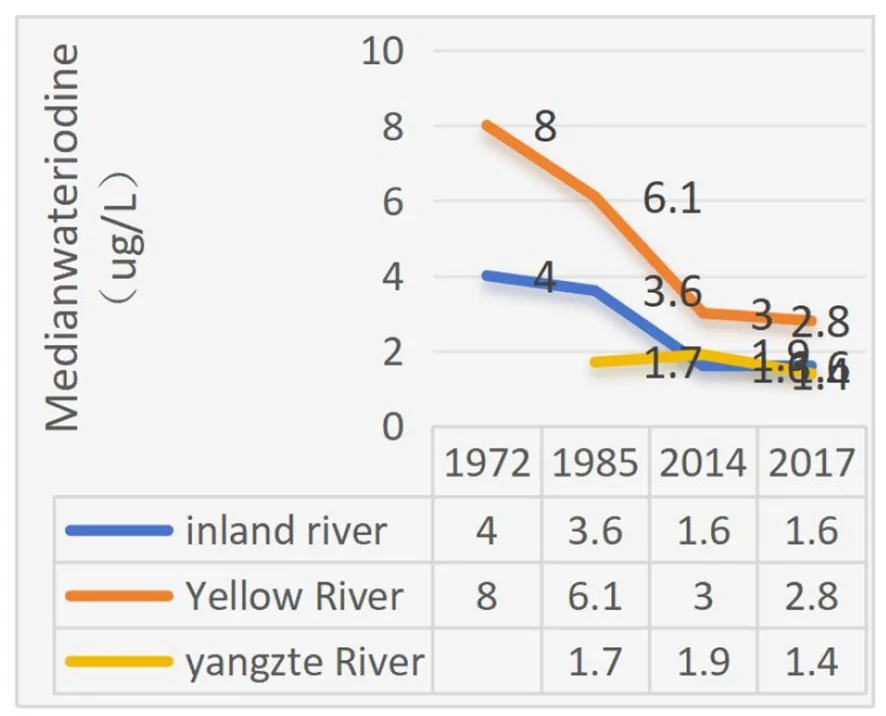
Trends in water iodine in different basins.

Notably, the water iodine distributions in 2014 and 2017 showed substantial differences that warrant caution in interpretation. For example, in the Inland River Basin, 21.7% of samples fell in the 20–30 μg/L range in 2014, whereas none fell in this range in 2017. In the Longdong region, the median decreased from 10.5 μg/L in 2014 to 5.8 μg/L in 2017—a 44.8% reduction over 3 years. Upon re-examination of our survey records, we found that the 2014 and 2017 surveys used different sampling site coverage; the 2014 survey included a higher proportion of villages with naturally elevated iodine levels that were not revisited in the 2017 survey. Therefore, these differences likely reflect sampling heterogeneity rather than rapid environmental change. Supplementary sensitivity assessment: if high-iodine subsamples (20–40 μg/L range in 2014) are hypothetically excluded, the overall median magnitudes across watersheds shift moderately, confirming that inclusion of high-iodine villages substantially drives the between-survey discrepancy. No genuine short-term environmental iodine drop over 2014-2017 can be concluded.

Table 2 shows the median iodine in Longnan water was 2.6, 2.0, and 1.3 μg/L for the three tests in 1985, 2014, and 2017, respectively, The median iodine in Hexi was 4.0, 3.6, 1.6 and 1.6 μg/L, respectively, The median iodine in Longzhong water was 2.0, 6.7, 2.3 and 2.2μg/L, respectively, The median iodine in Longdong water was 8.0, 8.8, 10.5 and 5.8μg/L respectively, The 4-years median water iodine in Longnan, Hexi, Longzhong and Longdong were all significantly different (all *P* < *0.05*, after Bonferroni correction; see Table 2 footnotes for detailed test statistics); As shown in Figure 3, during the same period, the median water iodine in the four regions were significantly different (all *P*<*0.05*; see Table 2 footnotes for detailed test statistics), presenting a consistent trend of Longdong > Longzhong > Hexi > Longnan.

**Table 2:** Water iodine content in different landforms of Gansu Province.

Watershed	Year	Number of copies	M (*P*_25_ ~*P*_75_) (μg/L)	Iodine frequency distribution [***n*** (%)]					Significance test	
				0~	10~	20~	30~	40~	χ^2^	*P*
Hexi	1972	1835	4.0 (2.0 ~ 8.0)	1525 (83.1)	222 (12.1)	40 (2.2)	17 (0.9)	31 (1.7)	63.309	0.000
	1985	49	3.6 (2.7 ~ 6.6)	42 (85.7)	5 (10.2)	2 (4.1)	0 (0.0)	0 (0.0)		
	2014	315	1.6 (0.5 ~ 11.2)	232 (73.7)	17 (5.4)	65 (20.6)	0 (0.0)	1 (0.3)		
	2017	242	1.6 (0.7 ~ 3.2)	236 (97.5)	6 (2.5)	0 (0.0)	0 (0.0)	0 (0.0)		
Longzhong	1972	61	2.0 (1.0 ~ 7.0)	50 (82.0)	11 (18.0)	0 (0.0)	0 (0.0)	0 (0.0)	13.251	0.004
	1985	40	6.7 (2.6 ~ 13.2)	26 (65.0)	9 (22.5)	3 (7.5)	1 (2.5)	1 (2.5)		
	2014	232	2.3 (0.6 ~ 3.8)	201 (86.6)	28 (12.1)	1 (0.4)	2 (0.9)	0 (0.0)		
	2017	447	2.2 (1.0 ~ 3.1)	428 (95.7)	12 (2.7)	2 (0.4)	1 (0.2)	4 (0.9)		
Longdong	1972	1255	8.0 (4.0 ~ 10.0)	732 (58.3)	454 (36.2)	11 (0.9)	7 (0.6)	51 (4.1)		0.000
	1985	23	8.8 (4.2 ~ 11.4)	14 (60.9)	9 (39.1)	0 (0.0)	0 (0.0)	0 (0.0)	42.062	
	2014	187	10.5 (4.9 ~ 32.3)	82 (43.9)	90 (48.1)	14 (7.5)	0 (0.0)	1 (0.5)		
	2017	231	5.8 (3.6 ~ 8.2)	200 (86.6)	26 (11.3)	4 (1.7)	1 (0.4)	0 (0.0)		
Longnan	1985	42	2.6 (1.4 ~ 3.4)	42 (100.0)	0 (0.0)	0 (0.0)	0 (0.0)	0 (0.0)	21.295	0.000
	2014	467	2.0 (0.0 ~ 4.1)	424 (90.8)	40 (8.6)	1 (0.2)	2 (0.4)	0 (0.0)		
	2017	265	1.3 (0.8 ~ 3.3)	259 (97.7)	5 (1.9)	0 (0.0)	1 (0.4)	0 (0.0)		

**Figure 3:**
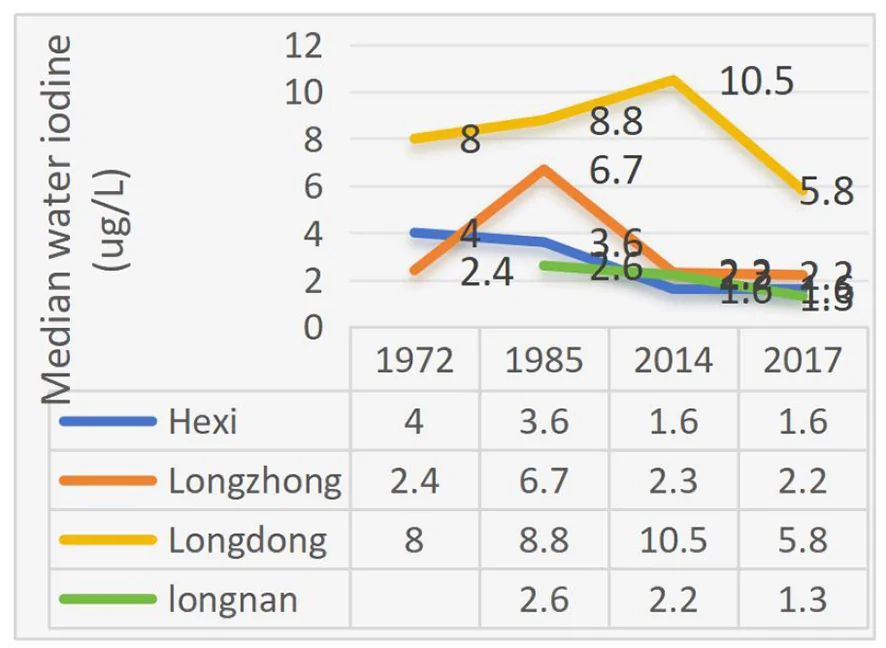
Trends in water iodine in different landscapes.

### 3.2 Soil iodine levels

#### 3.2.1 Soil iodine content in different watersheds

Table 3 shows the median soil values for the inland river, the Yellow River and the Yangtze River were 58.8 mg/kg, 11.5 mg/kg, and 33.8 mg/kg in 1985, respectively.8.6 mg/kg, 12.8 mg/kg and 16.3 mg/kg in 2014, respectively, Soil iodine in the inland river and Yangtze River Basin was significantly lower than that in 1985 (*Z*_*inland*_ = −*9.100, Z*_*YangtzeRiver*_ = *2.264, both P* < *0.05*), especially in the western part of the Hexi region where soil iodine was reduced from 58.8 mg/kg to 8.6 mg/kg, while the iodine level in the Yellow River Basin did not change significantly; The difference in soil iodine content among the three watersheds during the same period was significant ($\chi_{1985}^{2}$ = *61.938*, $\chi_{2014}^{2}$ = *40.387*, both *P* < *0.05*), with soil iodine inland river > Yangtze River > Yellow River in 1985, and Yangtze River > Yellow River> inland river in 2014 (Figure 4). Notably, soil iodine median in Yellow River Basin slightly increased from 11.5 mg/kg (1985) to12.8 mg/kg (2014), though without statistical significance (*P* > 0.05). This non-significant opposite trend relative to inland-river basin highlights heterogeneous soil-iodine trajectories across basins.

**Table 3:** Comparison of soil iodine content in different watersheds.

Land form type	Year	Number of copies	M (*P*_25_ ~*P*_75_) (mg/kg)	Frequency distribution of soil iodine content *n* (%)				Significance test	
				0~	5~	10~	20~	Z	*P*
Inland river	1985	64	58.8 (35.1 ~ 100.0)	0 (0.0)	0 (0.0)	4 (6.3)	60 (93.7)	−9.100	0.000
	2014	50	8.6 (5.4 ~ 11.5)	12 (24.0)	19 (38.0)	19 (38.0)	0 (0.0)		
Yellow river	1985	71	11.5 (5.4 ~ 24.0)	16 (22.5)	13 (18.3)	20 (28.2)	22 (31.0)	0.157	0.692
	2014	50	12.8 (9.8 ~ 17.6)	3 (6.0)	11 (22.0)	28 (56.0)	8 (16.0)		
Yangtze river	1985	35	33.8 (15.7 ~ 462.5)	0 (0.0)	4 (11.4)	6 (17.1)	25 (71.4)	2.264	0.000
	2014	55	16.3 (12.7 ~ 21.8)	2 (3.6)	7 (12.7)	26 (47.3)	20 (36.4)		

**Figure 4:**
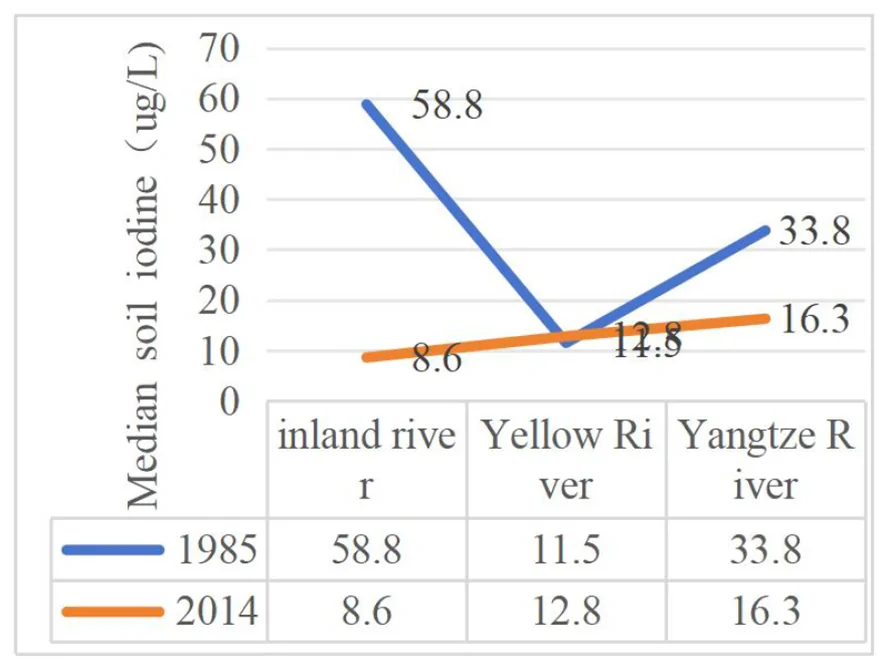
Trends in soil iodine in different landscapes.

#### 3.2.2 Iodine content of soils in different landscapes

Table 4 shows in two soil tests in 1985 and 2014, the median soil values were 30.9, 58.8, 23.0 and 11.8 mg/kg in Longnan, Hexi, Longzhong and Longdong soils in 1985, respectively, The median soil values in 2014 were 16.3, 8.6, 17.6 and 10.8 mg/kg, respectively, Soil iodine in Longnan and Hexi was significantly lower in 2014 than in 1985 (Z_Longnan_ = 3.011, Z_Hexi_ ~ = −9.100, both *P* < 0.05), especially in Hexi, where soil iodine decreased from 58.8 mg/kg in 1985 to 8.6 mg/kg in 2014; The soil iodine content of the four districts differed significantly in the same period ($\chi_{1985}^{2}$ = *42.847*, $\chi_{2014}^{2}$ = *38.831, both P* < *0.05*), soil iodine Hexi > Longnan > Longzhong > Longdong in 1985, and Longzhong > Longnan > Longdong > Hexi in 2014. Figures 5 shows the trends in soil iodine in different landscapes. Marked re-ranking of regional soil-iodine medians occurred between 1985 and 2014, indicating large-magnitude temporal shifts in spatial patterns of top-soil iodine across Gansu.

**Table 4:** Soil iodine content in different landscapes.

Landform type	Year	Number of copies	M (*P*_25_ ~*P*_75_) (mg/kg)	Frequency distribution of soil iodine content *n* (%)				Significance test	
				0~	5~	10~	20~	Z	*P*
Longnan	1985	40	30.9 (11.3 ~ 462.5)	0 (0.0)	7 (17.5)	8 (20.0)	25 (62.5)	3.011	0.003
	2014	55	16.3 (12.7 ~ 21.8)	2 (3.6)	7 (12.7)	26 (47.3)	20 (36.4)		
Hexi	1985	64	58.8 (35.1 ~−100.0)	0 (0.0)	0 (0.0)	4 (6.3)	60 (93.7)	−9.100	0.000
	2014	50	8.6 (5.4 ~ 11.5)	12 (24.0)	19 (38.0)	19 (38.0)	0 (0.0)		
Longzhong	1985	35	23.0 (1.7 ~ 31.5)	16 (45.7)	0 (0.0)	0 (0.0)	19 (54.3)	−0.079	0.937
	2014	20	17.6 (14.0 ~ 20.9)	0 (0.0)	2 (10.0)	12 (60.0)	6 (30.0)		
Longdong	1985^*^	31	11.8 (7.2 ~ 14.8)	0 (0.0)	10 (32.3)	18 (58.1)	3 (9.7)	−0.550	0.583
	2014^*^	30	10.8 (8.0 ~ 13.3)	3 (10.0)	9 (30.0)	16 (53.3)	2 (6.7)		

**Figure 5:**
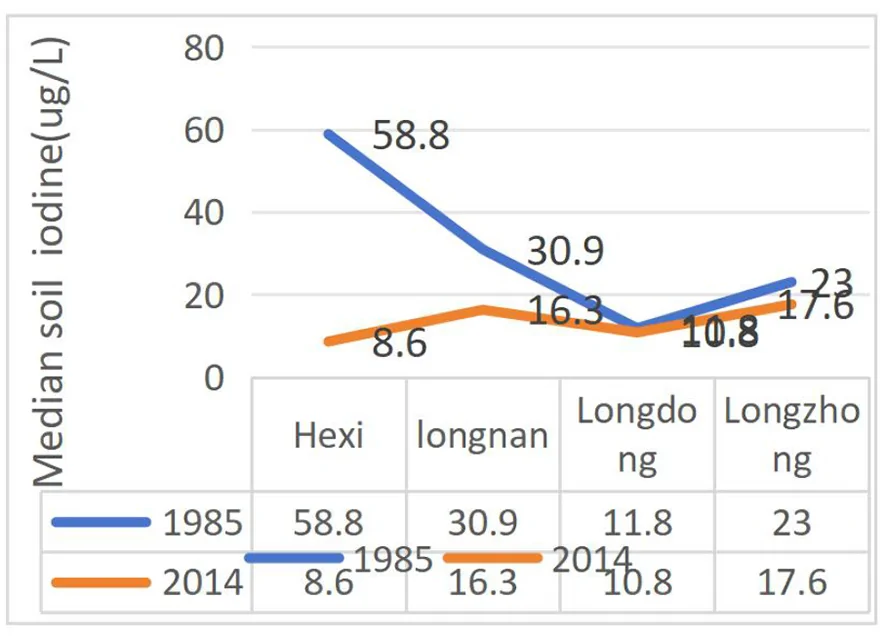
Trends in soil iodine in different landscapes.

## 4 Discussion

The oceans constitute the primary global reservoir of iodine, and iodine maintains its biogeochemical equilibrium through both marine and terrestrial food chains. Iodine enters the oceans via continental runoff, is released into the atmosphere under solar radiation, and returns to land through precipitation, thereby perpetuating a natural iodine cycle. Gansu Province encompasses three major river basins—the Yellow River, the Yangtze River, and the Inland River—and four distinct geomorphological regions: Longdong, Longzhong, Hexi, and Longnan. Longdong and Longzhong belong to the Yellow River basin, Hexi belongs to the Inland River basin, and Longnan belongs to the Yangtze River basin. Across the four survey periods, water iodine concentrations consistently followed the order: Yellow River basin > Inland River basin > Yangtze River basin, and Longdong > Longzhong > Hexi > Longnan, indicating that the spatial distribution of water iodine was coherent at both watershed and regional scales. Surface water iodine levels can vary substantially during rainfall events (11). Multi-year water resource monitoring in Gansu has shown that total precipitation follows the same sequence: Yellow River basin > Inland River basin > Yangtze River basin (12), suggesting a positive association between precipitation and water iodine concentrations.

This pattern of low iodine concentrations in mountainous and inland basins is not unique to Gansu. Globally, approximately 1.9 billion people live in iodine-deficient environments, and similar spatial distributions have been documented elsewhere. For example, groundwater iodine in the Gandak basin of Bihar, India—a region reliant solely on groundwater for drinking—ranged from 2.1 to 4.56 μg/L, confirming persistent deficiency and a high prevalence of endemic goiter in that alluvial plain (13). Likewise, soil surveys in the Lower Himalayan region of Pakistan revealed significantly lower iodine concentrations in mountainous areas than in adjacent plains, with soil iodine positively correlated with organic matter and clay content (14). These international observations indicate that the hydro-geomorphic control observed in Gansu—i.e., lower iodine in headwater and arid basins—is a globally recurring phenomenon, reinforcing the representativeness of our results.

Against the backdrop of regional climate warming across Gansu (15, 16, 36), precipitation exhibits complex spatial heterogeneity—increasing in western Gansu and decreasing in southeastern parts (17–20) rather than showing a province-wide uniform decline. The water iodine patterns across basins and regions exhibited consistent temporal differences among the survey years. Soil iodine also showed temporal variations, and the spatial distribution across watersheds and regions remained consistent within each survey year. However, after 30 years of environmental change, the regional pattern shifted markedly: in 1985, the highest soil iodine was found in the Hexi region (Inland River basin), whereas by 2014, Hexi had the lowest value; soil iodine in Longnan (Yangtze River basin) decreased moderately, while that in the Yellow River basin showed no significant change. The factors controlling soil iodine are complex (21). On one hand, soil organic matter strongly adsorbs and fixes iodine; higher organic matter content favors iodine retention and reduces its transport and loss (4). The vegetation (organic matter) cover in Hexi, Longzhong, Longdong, and Longnan ranked from poor, low, moderate, to high, respectively (22), meaning that iodine adsorption is weakest in Hexi soils. On the other hand, precipitation acts primarily as a leaching agent for soil iodine rather than a replenishing source (23); increased rainfall can reduce soil iodine via vertical percolation (24). Hexi and Longnan experience higher temperatures than Longzhong and Longdong (15). Precipitation patterns vary considerably between western and central-eastern areas, with a general increase in the west and a decrease in the southeast (17–20). Rising temperatures may increase glacial meltwater discharge from the Qilian Mountains. We speculate that combined stressors including temperature regime, precipitation patterns and low vegetation cover jointly contribute to top-soil iodine depletion in Hexi, yet direct local empirical evidence is lacking (25, 26). In contrast, Longnan (Yangtze basin) and the Yellow River basin (Longdong and Longzhong) have relatively better vegetation cover and reduced rainfall, resulting in less soil iodine loss. Our findings are consistent with the hypothesis that glacial dissolution and water scouring contribute to terrestrial iodine deficiency (1); one speculative scenario is that enhanced glacial melt and water erosion may remove iodine-rich topsoil; however, this mechanism lacks field-scale evidence within Gansu, and the newly formed soils from parent rock contain only about one-quarter of the original iodine content.

We should note that such an 85% magnitude drop in Hexi top-soil iodine could partially originate from methodological inconsistencies across two historical soil surveys, including differences in archived field sampling operations, sample pre-treatment, or analytical performance, not exclusively real environmental loss.

The mechanisms underlying these soil iodine changes are further illuminated by international geochemical studies. Shetaya et al. examined the adsorption kinetics of iodine (^129^I) in nine soils of contrasting properties and found that iodide removal from solution was extremely rapid (minutes to hours) while iodate removal was slower (hours to days); importantly, phosphate-extractable inorganic iodine was very low, indicating that inorganic sorption contributes little to iodine incorporation into humic substances, and that iodine retention is strongly enhanced by high organic carbon, low pH, and elevated temperature (27). This mechanistic insight helps explain the sharp decline in Hexi: poor vegetation and low organic matter weaken iodine fixation, while rising temperatures and increased glacial meltwater accelerate vertical leaching. In a long-term forest soil budget study in France, Roulier et al. demonstrated that throughfall accounted for 83 % of iodine inputs, litterfall only 17 %, and that soil iodine concentration is determined by the balance between inputs and retention capacity (linked to organic matter, iron, and aluminum); the residence time of iodine in those soils was estimated at 419–1756 years (28). This underscores the critical role of vegetation cover and soil properties in iodine retention—consistent with our observation that Longnan, with its higher vegetation cover, experienced much less soil iodine loss. Additionally, research in the Lower Himalayas attributed soil iodine depletion to erosion and excessive precipitation-induced leaching (14). Collectively, these international data support our conclusion that the pronounced soil iodine reduction in Hexi results from the combined effects of climatic warming, glacial meltwater increase, and a fragile ecological environment with low organic matter and vegetation cover.

A critical finding that warrants careful interpretation is the apparent divergence between water and soil iodine responses to precipitation. At the watershed scale, water iodine was higher in areas with greater precipitation (Yellow River basin), suggesting a positive association between rainfall and surface water iodine. In contrast, our soil data indicated that precipitation primarily acts as a leaching agent, reducing soil iodine through vertical percolation—a mechanism widely documented (19, 20). We propose that these two observations are not contradictory but reflect different hydrological processes operating in different compartments of the iodine cycle. Precipitation leaches soluble iodine from rocks and soils in the upper catchment, mobilizing it into the river network, thereby increasing water iodine concentrations in wetter basins. Simultaneously, the same precipitation drives vertical leaching through the soil profile, removing iodine from the topsoil. Thus, high-rainfall areas may simultaneously exhibit higher water iodine (due to lateral mobilization) and lower topsoil iodine (due to vertical leaching)—a phenomenon we term “hydrologic decoupling.”

This decoupling has been observed elsewhere. For instance, the iodine mass balance in Lake Constance (Germany) showed that alpine tributaries with higher glacial meltwater fractions had lower iodine concentrations (1–2 μg/L) in summer, while lowland rivers had higher values (2–10 μg/L), and organic-bound iodine accounted for 64 % of total dissolved iodine inputs (29). Moreover, Roulier et al. explicitly showed that leaching of iodine-bearing organic compounds from forest soils is a major source of dissolved organic iodine in groundwater and streams (28). These findings confirm that precipitation simultaneously enriches surface waters and depletes topsoils. However, we acknowledge that this conceptual framework, while plausible, remains speculative without paired water-soil sampling at identical locations across years. Alternative factors—such as differential fertilizer application, changes in irrigation intensity, or water conservancy projects—could also contribute (25, 26). Importantly, recent reviews have highlighted that climate change increasingly perturbs the iodine biogeochemical cycle. Smyth et al. noted that global warming may enhance atmospheric iodine production via ozone-mediated volatilisation from oceans and macroalgae, yet in arid and semi-arid regions, rising temperatures and altered precipitation more directly reduce soil iodine availability by accelerating organic matter decomposition and enhancing leaching losses (30). This global perspective reinforces our hypothesis that the soil iodine decline in Hexi is part of a broader climate-sensitive process. Future studies integrating atmospheric iodine measurements would be particularly valuable to fully validate this framework.

The substantial discrepancies between the 2014 and 2017 water iodine surveys—especially the disappearance of high-value outliers (20–30 and 30–40 μg/L ranges) within just three years—are unlikely to reflect genuine environmental change. These discontinuities are more plausibly attributable to differences in sampling site coverage: the 2014 survey may have inadvertently included a higher proportion of villages with naturally elevated iodine levels that were not revisited in 2017. This underscores a fundamental limitation of comparing independently designed surveys without fixed monitoring stations. The 2014 and 2017 surveys used different sampling site coverage, which may have introduced sampling heterogeneity, limiting our ability to interpret these differences as true temporal trends. Future surveys with fixed monitoring sites are needed to validate these observations.

Despite these methodological limitations, our findings have several practical implications for IDD prevention in Gansu Province:

(1) Maintain universal salt iodisation (USI): persistently low drinking-water iodine (well below WHO 10-20 μg/L range) confirms environmental iodine deficiency across Gansu. We recommend sustaining current USI dosage; any adjustment of salt-iodine concentration should await integrated monitoring combining soil iodine, local food iodine and population urinary iodine biomarkers, rather than based solely on soil-iodine data.
(2) Targeted monitoring in Hexi: given the sharp observed top-soil-iodine reduction in Hexi, we recommend implementing surveillance chains: local crop/food iodine content → population urinary iodine status, to judge whether environmental changes translate into population nutrition risks.
(3) Establish atmospheric iodine monitoring: a critical data gap is the absence of atmospheric iodine measurements. We recommend establishing a pilot atmospheric monitoring station in Gansu to enable future integration of air-water-soil iodine data and to elucidate the complete biogeochemical cycle.
(4) Adopt standardized fixed-site surveys: Future surveys should use fixed monitoring sites and standardized analytical methods to enable valid longitudinal comparisons and to eliminate the methodological uncertainties inherent in historical cross-sectional datasets. Such surveys would provide a robust evidence base for future policy refinements.

Our recommendations are consistent with global public health perspectives. Universal salt iodisation (USI) has been endorsed by the World Health Organization as the most cost-effective strategy for controlling IDD and is currently implemented in over 120 countries. Horton and Miloff (2010), in a cross-country analysis, demonstrated that median urinary iodine concentrations are positively associated with household availability of iodised salt (elasticity 0.73–0.83), and that soil iodine content also exerts a significant positive effect on population iodine status (31). However, the field of medical geology reminds us that despite the remarkable success of USI, persistent environmental iodine deficiency means that salt iodisation alone may not fully address all challenges, particularly in regions with high rainfall and leaching losses where environmental iodine vulnerability is elevated. Such considerations suggest that, in addition to maintaining USI, targeted environmental surveillance and, where feasible, complementary interventions (e.g., iodine-fortified irrigation water or fertilizers in agricultural areas) could be considered in the most depleted zones (32). These international experiences reinforce our proposed strategy for Gansu: maintain USI, but strengthen environment-based monitoring to adapt to local changes.

We emphasize that these recommendations are provisional and should be reassessed as higher-quality data become available through future standardized monitoring programs. In summary, iodine concentrations in the external environment of Gansu Province have remained low across all surveys, with observed temporal fluctuations that should be interpreted with caution due to the methodological limitations discussed above. According to national criteria (33, 34), the study area is classified as iodine-deficient, with only a few scattered villages having adequate iodine levels and no areas with excessive iodine intake. There is a point distribution of suitable iodine intake, but no high iodine intake. Iodine is distributed in water, soil, and air. However, the absence of atmospheric iodine measurements in this study prevents us from assessing the atmospheric component of the iodine cycle. International geochemistry has long recognized that the majority of terrestrial iodine originates from the atmosphere, which in turn receives iodine from marine emissions; the ocean-to-atmosphere-to-land pathway is the dominant driver of the global iodine cycle (36). Yet quantitative data on atmospheric iodine fluxes remain scarce, especially in inland regions. Future research should prioritize atmospheric iodine monitoring to elucidate the complete air-water-soil iodine dynamics. Iodine in atmospheric precipitation is higher on the coast than on the mainland in China (35). In this study, only water and soil iodine were observed; further testing of atmospheric iodine is needed to clarify the state of natural iodine cycling and characterize ecological changes.

Several important limitations must be considered. First, the four water surveys (1972, 1985, 2014, 2017) and two soil surveys (1985, 2014) were not designed as longitudinal monitoring programs with fixed sampling sites. Sampling locations varied across survey years, and sample sizes differed substantially (e.g., Yellow River: *n* = 1,316 in 1972 vs. *n* = 805 in 2017; Inland River: *n* = 1,835 in 1972 vs. *n* = 49 in 1985). Consequently, the observed differences between years may partially or fully reflect sampling heterogeneity, geographic coverage differences, and methodological variability rather than purely environmental changes. We have therefore deliberately avoided using definitive causal language (e.g., “decline”) when describing temporal patterns and instead present our findings as observational cross-sectional patterns that generate hypotheses for future investigation. Second, methodological details for the earliest surveys (1972, 1985) are incompletely documented in the original archives. Although we have provided the available protocols in Section 2.2, we cannot exclude the possibility of analytical drift or changes in detection limits over the four decades that might affect comparability. Third, the coarse spatial resolution of our analysis (watershed and regional averages) obscures within-area heterogeneity and precludes fine-scale geostatistical analysis. Fourth, we lacked atmospheric iodine measurements, which prevented us from fully characterizing the complete air-water-soil iodine cycle. Future studies with standardized, fixed-site monitoring programs integrating all three environmental compartments are urgently needed to validate and extend our observational findings.

## Data Availability

The raw data from the four historical surveys are not publicly available due to institutional data protection policies, but all summary statistics (medians, percentiles, sample sizes, and frequency distributions) are fully reported in the tables. Researchers may contact the corresponding author (Ye Ruan, ruany1203@163.com) for reasonable data access requests, subject to approval by the Gansu Provincial Center for Disease Control and Prevention.
